# Association between intestinal microflora and renal function in patients with chronic renal failure: A case-control analysis

**DOI:** 10.12669/pjms.40.1.8194

**Published:** 2024

**Authors:** Haixia Chen, Xiaoxiao Xie, Shuming Tang

**Affiliations:** 1 Haixia Chen, Department of Clinical Laboratory, Shenzhen People’s, Hospital the Second Clinical Medical College, Jinan University, The First Affiliated Hospital, Southern University of Science and Technology, Shenzhen, Guangdong Province 518020, China; 2 Xiaoxiao Xie, Department of Clinical Laboratory, Shenzhen People’s, Hospital the Second Clinical Medical College, Jinan University, The First Affiliated Hospital, Southern University of Science and Technology, Shenzhen, Guangdong Province 518020, China; 3 Shuming Tang, Department of Clinical Laboratory, Shenzhen People’s, Hospital the Second Clinical Medical College, Jinan University, The First Affiliated Hospital, Southern University of Science and Technology, Shenzhen, Guangdong Province 518020, China

**Keywords:** Chronic renal failure, Intestinal microflora, Structural change, Renal function, GFR, BUN, SCr, CysC

## Abstract

**Objective:**

To identify the association between the changes in intestinal microflora and renal function in patients with chronic renal failure (CRF).

**Methods:**

This retrospective case-control study included 50 patients with CRF (study group), admitted to the Clinical Laboratory Department of Shenzhen People’s Hospital from March 2021 to May 2022, and 50 healthy individuals (control group). The association between the distribution of intestinal microflora and the glomerular filtration rate (GFR), levels of serum creatinine (SCr), blood urea nitrogen (BUN), and serum cystatin C (CysC) were analyzed.

**Results:**

Intestinal microflora of CRF patients had significantly higher levels of Enterococci compared to the control group (p-Value <0.05), while the levels of Bifidobacterium spp. and Escherichia coli were lower in the study group (p-Value <0.05). GFR was lower, and the levels of BUN, SCr, and CysC were higher in the study group compared to the control group (all p-Value <0.05). GFR, BUN, SCr and CysC levels in the study group negatively correlated with the levels of Bifidobacterium spp. and Lactobacillus spp. (r<0, P<0.05), and positively correlated with the abundance of Enterococcus spp. and Escherichia coli (r>0, P<0.05) in the intestinal microflora.

**Conclusions:**

Changes in intestinal microbiota are associated with a significant decrease in GFR and a marked increase in serum levels of renal function indicators, and alterations in the balance of intestinal microbiota may lead to further aggravation of the renal function damage in patients with CRF.

## INTRODUCTION

Chronic renal failure (CRF) occurs with a high incidence, affecting more than 10% of the world’s population, and is caused by a variety of conditions, such as nephritis, polycystic kidney disease, and nephrotic syndrome.^1^,^2^ The disease is characterized by the severe destruction of the renal parenchyma and continuous deterioration of renal function.^3^ Numerous studies show that the intestinal microbiota directly participates in various physiological processes including metabolism, vitamin synthesis, regulation of intestinal mucosal immunity, and nutrient absorption.^4^ The interaction between the immune system and the normal intestinal microflora can effectively prevent invasion by pathogenic bacteria.^4^,^5^

Furthermore, dominant populations of symbiotic bacteria in the intestinal microflora can effectively inhibit colonization with pathogenic bacteria.^5^ Changes in the intestinal microflora may lead to immune system abnormalities that can result in disease.^3^,^5^ Intestinal microflora imbalance, or dysbiosis, may be caused by a number of factors, such as the use of antibiotics, stress, age, dietary habits and lifestyle.^6^ Dysbiosis in patients with CRF is associated with dietary restrictions, the use of specific medications, and infiltration of urinary toxins into the intestinal cavity.^7^ Moreover, studies show that the occurrence and progression of CRF directly correlate with the microecological changes in the intestinal microflora.^8^

The results of animal studies conducted by Bakris GL et al.^9^ showed that the intestinal permeability in CRF rats significantly increased with the increase in the extent of the intestinal injury. The impact of the changes in the composition of the intestinal microflora on the metabolism of nutrients may result in further worsening of metabolic disorders that can ultimately lead to CRF aggravation.^8^,^9^ There have been many studies on gastrointestinal microbiota in patients with chronic kidney disease, but few studies explore the association between gastrointestinal microbiota changes and renal function in patients with CRF.^10^,^11^ Therefore, the aim of this study was to evaluate the association between the changes in intestinal microflora and renal function indicators, such as glomerular filtration rate (GFR), levels of serum creatinine (SCr), blood urea nitrogen (BUN), and serum cystatin C (CysC), in patients with CRF. The results of our study may further help to gain insights into the pathogenesis of CRF.

## METHODS

This was a retrospective case-control study. The clinical data of the patients with CRF diagnosed at the Clinical Laboratory Department of Shenzhen People’s Hospital from March 2021 to May 2022 were reviewed and finally 50 patients were screened as the study group, while the control group included 50 healthy individuals observed during the same period. The groups were matched for age and gender.

### Inclusion criteria:

- The diagnoses of individuals in the study group met the clinical diagnostic criteria for CRF.^12^
- Individuals in the control group were healthy according to the results of physical examination.
- All participants had normal cognitive and mental functions.
- All participants were ≥ 18 years.
- CCr < 80 mL/min.
- GFR< 60 mL/min/1.73m^2^, and last for >3 months.
- Complete relevant clinical data.

### Exclusion criteria:

- Patients with infectious diseases, such as viral hepatitis, AIDS, pneumonia, or the new coronavirus.
- Patients with malignancies.
- Immune system diseases, including autoimmune diseases, immunodeficiency diseases, and allergic diseases.
- Individuals with serious conditions affecting heart, lung, or stomach function, and patients with hepatobiliary disease.
- Individuals with severe coagulation dysfunction.
- Patients with severe hydronephrosis.
- Patients with intestinal infections during that occurred within three months before the study.
- Individuals using prebiotics, probiotics, or antibiotics.

### Ethical Approval

The Medical Ethics Committee of our hospital approved this study (No. LL-KY-2019424, Date: 2019-07-25).

All participants provided fecal samples to assess the composition of their intestinal microflora. Specimens were collected by sterile collectors and sterile toothpicks were used to take the mid-section of the samples to prevent cross-contamination. Two weighed fecal samples were dissolved in 4mL of normal saline each, and then diluted to 10^-6^ concentrations before being inoculated using a drip method. The following growth media were used for culturing: Bifidobacterium-BS, Lactobacillus-LBS, Escherichia coli-EMB, and Enterobacterium-TTC agar plates (Guangdong Huankai Microbiology Technology, China). Fecal samples were cultured in triplicates for each dilution. BS and LBS agar plates were placed in an anaerobic incubator with a temperature of 37°C for 48 hours, and EMB and TTC agar plates were placed in an aerobic biochemical incubator at 37°C for 24 hours. After the incubation period, shape, color, size, edge, and density of the colonies on the plates was observed a representative colony was selected for Gram staining, and the microorganisms were visualized using a light microscope (Nikon ECLIPSE Ni-U, Japan).

On the aerobic EMB agar plates, Escherichia coli appeared as round colonies with neat edges and smooth or slightly convex surfaces. Under the light microscope, the bacteria appeared as straight rods in pairs. Lactobacillus spp. grew on anaerobic agar LBS plates as round gray or white colonies, 0.5-2.0-mm in diameter, with regular edges, and a smooth surface. The bacteria appeared as elongated bacilli arranged in single short chains and grids. The Enterococcus spp. on the aerobic agar TTC plate presented as round, rough, flat, dark red colonies with a diameter of 0.5-1.5 mm and a lack of neat edges. Under the light microscope, the Gram-positive bacilli appeared oval or spherical (a few were globular) arranged in pairs, long or short chains, or bundles. Bifidobacterium spp. grew on the anaerobic agar BS plates and presented as round, gray and white translucent colonies of neat edges, smooth surface, and 0.7-2.4 mm in diameter. Under the light microscope, these Gram-positive bacteria had uniform cell color cells of diverse shapes (mainly curved, straight, bifurcated, or rod-shaped, without flagella). Final number of bacteria were calculated as the number of colonies per unit weight.

We collected 5 mL of fasting venous blood from each participant to measure levels of renal function indicators. Briefly, samples were centrifuged at 5000 r/min for eight minutes and the supernatants were used to measure SCr, BUN, and CysC levels using a fully automatic biochemical analyzer (model AU5800; Beckman Kurt, USA). BUN was detected via the urease-glutamate dehydrogenase method, with a reference range of 2.8 to 7.6 mmol/L. Reference ranges for SCr levels were 40 to 90 μmol/L for women and 64 to 104 μmol/L for men. Immunoturbidimetry was used for CysC detection, with a reference value range of 0.5 to 1.07 mg/L. We calculated the GFR using the CKD-EPI formula: 1.86 × SCr -1.164 × age -0.203.

### Statistical Analysis

Data was processed and analyzed using SPSS 25.0 software. Counting data were expressed as means and standard deviations (*χ̅*±*s*), and inter-group comparisons were done using one-way ANOVAs. All count data were expressed as percentages (n [%]) and inter-group comparisons were performed via *χ^2^* tests. Pearson correlation analysis was used for analyzing the correlation between intestinal microflora and renal function indicators. p-Value < 0.05 was considered as statistically significant.

## RESULTS

There was no difference in the mean basic characteristics between the two groups (p-Value >0.05; Table-I). The abundance of intestinal Lactobacillus spp. was comparable between the two groups (P>0.05). However, the abundance of Escherichia coli and intestinal Bifidobacterium spp. in the study group were significantly lower than in the control group (p-Value <0.05), while the level of Enterococcus spp. was significantly higher (p-Value <0.05; Table-II).

**Table-I:** Comparison of general data between the two study groups.

Group	n	Gender (male/female)	Age (years)	BMI (kg/m²)
Study-group	50	29/21	49.14±10.88	23.92±2.78
Control-group	50	32/18	50.96±10.36	24.61±2.40
*χ^2^*/*t*	-	0.378	-0.856	-1.338
*P*	-	0.539	0.394	0.184

**Table-II:** Comparison of intestinal flora between the two study groups (lg CFU/g,*χ̅*±*s*).

Group	n	Enterococcus spp.	Bifidobacterium spp.	Escherichia coli spp.	Lactobacillaceae spp.
Study-group	50	8.66±2.03	4.88±1.01	3.95±1.10	6.58±1.49
Control-group	50	5.74±1.49	7.62±2.01	6.28±1.62	6.81±1.54
*t*		8.180	-8.595	-8.392	-0.758
*P*		<0.001	<0.001	<0.001	0.450

Glomerular filtration rate of patients in the study group was significantly lower, and the levels of SCr, BUN and CysC were significantly higher than those in the control group (P<0.05; Table-III). The levels of GFR, SCr, BUN and CysC in patients with CRF negatively correlated with the intestinal levels of Bifidobacterium spp. and Lactobacillus spp. (r<0, P<0.05), and positively correlated with the abundance of Escherichia coli and Enterococcus spp. (r>0, P<0.05; Table-IV).

**Table-III:** Comparison of renal function indexes between the two groups (*χ̅*±*s*).

Group	n	GFR (ml/min)	SCr (μmol/L)	BUN (mmol/L)	CCysC (mg/L)
Study-group	50	16.02±3.03	385.32±28.05	23.42±3.29	4.95±1.44
Control-group	50	93.00±11.70	74.96±10.59	4.52±1.18	1.90±0.36
*t*	-	-45.040	73.192	38.234	14.477
*P*	-	<0.001	<0.001	<0.001	<0.001

**Table-IV:** Correlation between intestinal flora distribution and renal function in patients with CRF.

Intestinal flora	GFR		SCr		BUN		CysC	

	r	P	r	P	r	P	r	P
Enterococcus spp.	0.848	<0.001	0.843	<0.001	0.878	<0.001	0.829	<0.001
Bifidobacterium spp.	-0.905	<0.001	-0.837	<0.001	-0.860	<0.001	-0.862	<0.001
Escherichia coli	0.808	<0.001	0.831	<0.001	0.842	<0.001	0.811	<0.001
Lactobacillaceae spp.	-0.825	<0.001	-0.784	<0.001	-0.849	<0.001	-0.769	<0.001

r: Pearson correlation coefficient.

## DISCUSSION

This study showed that CRF was associated with markedly lower levels of intestinal Escherichia coli and Bifidobacterium spp., whereas the abundance of Enterococci was significantly higher, compared to healthy individuals. Serum levels of SCr, BUN, and CysC in patients with CRF were significantly higher and the GFR was significantly lower than in healthy individuals. Moreover, the levels of these renal function indicators in patients with CRF negatively correlated with the abundance of Lactobacillus spp. and Bifidobacterium spp., and positively correlated with the levels of Enterococcus spp. and Escherichia coli.

Intestinal microbiota is a complex ecosystem including more than 500 species of bacteria^13^,^14^ that maintain a symbiotic relationship with the human host and play a pivotal role in regulating carbohydrate metabolism and the immune system.^15^,^16^ Pasini E et al found that probiotic treatment in patients with chronic kidney disease can avert changes in the intestinal microflora composition, prevent enterogenous metabolic toxins from entering the circulation, and effectively inhibit the inflammatory response.^17^ Therefore, detecting these changes in intestinal microflora of patients with CRF and analyzing their potential correlations with the levels of renal function indicators may be important for disease prevention and treatment.^16^,^17^

Korpela K et al showed that the intestinal microflora of patients with kidney transplant or chronic kidney disease differs significantly from that of healthy individuals.^18^ Therefore, it is reasonable to hypothesize that patients with CRF may have abnormal intestinal microflora composition. Indeed, our study demonstrated that the abundance of Bifidobacterium spp., Escherichia coli, and Enterococcus spp. in patients with CRF differed significantly from that of healthy individuals. Sagiroglu G et al^19^ showed that the intestinal levels of Escherichia coli, Bifidobacterium spp. and Enterococcus spp. in patients with CRF were independent risk factors for the decrease in GFR. Our results further confirm this. We may hypothesize that the intestinal microflora of patients with CRF is mainly characterized by a decrease in the abundance of Escherichia coli and Bifidobacterium spp., and an increase in the abundance of Enterococcus spp.

Abnormal intestinal microflora in patients with CRF have been associated with the presence of intestinal endogenous toxins, such as p-cresol sulfate, indole sulfate, trimethylamine oxide, and lipopolysaccharide. Accumulation of these toxins was linked to the destruction of the intestinal barrier function, toxin invasion into the bloodstream, and the presence of symptoms of renal micro-and systemic inflammatory reactions that ultimately lead to the worsening of the renal function.^20^ Rubino CM et al. showed that the GFR of patients with CRF was significantly lower and the levels of CysC, BUN and SCr were significantly higher compared to healthy individuals, which is consistent with our results.^21^

Studies have also shown that the levels of BUN, CysC and SCr are higher in patients with severe CRF.^22^ Changes in the balance between the symbiotic and pathogenic bacteria can affect the intestinal barrier function, microecosystem stability, and ultimately damage renal function.^20^,^21^ BUN levels, which are significantly increased in all kinds of acute and chronic renal dysfunction, reflect the levels of urea nitrogen in the blood. SCr can be used to measure the glomerular filtration capacity, and is elevated in cases serious damage of the renal parenchyma. CysC concentration is more accurate for assessing glomerular filtration function.^22^ When the glomerular filtration function is slightly damaged, the concentration of CysC in the blood increases significantly and is proportional to the severity of the disease.^23^,^24^

In addition, the correlation analysis in this study showed that the levels of SCr, BUN, and CysC in patients with CRF significantly negatively correlated with the intestinal abundance of Lactobacillus spp. and Bifidobacterium spp., and significantly positively correlated with the intestinal levels of Enterococcus spp. and Escherichia coli. Taken together with previous observations, our results further support the perspective that intestinal microflora composition can affect renal function. Changes in intestinal microbiota in CRF patients, together with accumulating toxins, contribute to the severity of the disease, impair the intestinal barrier function, allow metabolites to enter the blood stream, and ultimately affect the renal function.^23^

Based on the findings above, fluctuations in intestinal microflora are shown to be linked to increased susceptibility to renal function damage. Therefore, it is suggested to pay attention to intestinal health and highlight the importance of intestinal microflora balance. Moreover, intestinal microflora may be potential targets in addressing CRF-related comorbidities, which may be a significant research direction in the future.

### Limitations of the study

It is a single-center retrospective study that relies on the accurate, detailed, and available diagnosis and treatment data of patients with CRF. We collected data from patients diagnosed and treated during 2022, and our sample size was relatively small. Further large-scale and multi-center studies are needed. Furthermore, intestinal microflora is a complex ecosystem containing hundreds of bacterial species, of which we only studied a few in this study.

## CONCLUSION

Changes in intestinal microbiota are associated with a significant decrease in GFR and a marked increase in serum levels of renal function indicators, and alterations in the balance of intestinal microbiota may lead to further aggravation of the renal function damage in patients with CRF.

### Authors’ contributions:

**HC:** Conceived and designed the study.

**XX and ST:** Collected the data and performed the analysis.

**HC:** Was involved in the writing of the manuscript and is responsible for the integrity of the study.

All authors have read and approved the final manuscript.
